# 2-Hydroxy-4-Methylbenzoic Anhydride Inhibits Neuroinflammation in Cellular and Experimental Animal Models of Parkinson’s Disease

**DOI:** 10.3390/ijms21218195

**Published:** 2020-11-02

**Authors:** Soo-Yeol Song, In-Su Kim, Sushruta Koppula, Ju-Young Park, Byung-Wook Kim, Sung-Hwa Yoon, Dong-Kug Choi

**Affiliations:** 1 Department of Biotechnology, Konkuk University, Chungju 380-701, Korea; go_world87@nate.com (S.-Y.S.); kis5497@kku.ac.kr (I.-S.K.); koppula@kku.ac.kr (S.K.); kbwxfile@nate.com (B.-W.K.); 2 Department of Molecular Science and Technology, Ajou University, Suwon 443-749, Korea; pink1209juyoung@empas.com (J.-Y.P.); shyoon@ajou.ac.kr (S.-H.Y.)

**Keywords:** microglia, neuroinflammation, nuclear factor kappa beta, interleukin, nitric oxide, Parkinson’s disease, 2-hydroxy-4-methylbenzoic anhydride (HMA)

## Abstract

Microglia-mediated neuroinflammation is one of the key mechanisms involved in acute brain injury and chronic neurodegeneration. This study investigated the inhibitory effects of 2-hydroxy-4-methylbenzoic anhydride (HMA), a novel synthetic derivative of HTB (3-hydroxy-4-trifluoromethylbenzoic acid) on neuroinflammation and underlying mechanisms in activated microglia in vitro and an in vivo mouse model of Parkinson’s disease (PD). In vitro studies revealed that HMA significantly inhibited lipopolysaccharide (LPS)-stimulated excessive release of nitric oxide (NO) in a concentration dependent manner. In addition, HMA significantly suppressed both inducible NO synthase and cyclooxygenase-2 (COX-2) at the mRNA and protein levels in LPS-stimulated BV-2 microglia cells. Moreover, HMA significantly inhibited the proinflammatory cytokines such as interleukin (IL)-1beta, IL-6, and tumor necrosis factor-alpha in LPS-stimulated BV-2 microglial cells. Furthermore, mechanistic studies ensured that the potent anti-neuroinflammatory effects of HMA (0.1, 1.0, and 10 μM) were mediated by phosphorylation of nuclear factor of kappa light polypeptide gene enhancer in B-cells inhibitor, alpha (IκBα) in LPS-stimulated BV-2 cells. In vivo evaluations revealed that intraperitoneal administration of potent neurotoxin 1-methyl-4-phenyl-1,2,3,6-tetrahydropyridine (MPTP, 20 mg/kg, four times a 1 day) in mice resulted in activation of microglia in the brain in association with severe behavioral deficits as assessed using a pole test. However, prevention of microglial activation and attenuation of Parkinson’s disease (PD)-like behavioral changes was obtained by oral administration of HMA (30 mg/kg) for 14 days. Considering the overall results, our study showed that HMA exhibited strong anti-neuroinflammatory effects at lower concentrations than its parent compound. Further work is warranted in other animal and genetic models of PD for evaluating the efficacy of HMA to develop a potential therapeutic agent in the treatment of microglia-mediated neuroinflammatory disorders, including PD.

## 1. Introduction

Lacking an effective treatment, increased environmental exposure of chemicals, and predisposing genetic factors, neurodegenerative diseases, have always been of prime interest to researchers. Various types of neurodegenerative disorders have their own specific set of neuronal circuitry that undergoes degeneration, resulting in specific disease symptoms [1,2,3]. Our body’s immune system has a sophisticated response to the dangers of internal or external stimuli [4,5]. However, sustained or prolonged immune response in the central nervous system (CNS) will ultimately cause excessive neuroinflammation, leading to damage to the adjacent neurons [3]. In case of an immune stimulus in the brain, microglial cells play a vital role and they are also the resident macrophages in the CNS [6]. Microglial cells have sensitive reactions (infectious diseases and oxidative stress) and high mobility [7]. These antigen-presenting cells, upon activation, produce various intermediate mediators (proinflammatory cytokines, chemokines, and reactive oxygen species) which are capable of destroying pathogens, but can also induce toxicity in neurons, leading to the occurrence of various inflammatory-mediated neurodegenerative disorders [8].

Mounting evidence suggests that neuroinflammation is one of the key mechanisms involved in acute brain injury and chronic neurodegeneration. In particular, microglia-mediated neuroinflammatory events are associated with pathogenesis of various neurodegenerative diseases including Parkinson’s disease (PD). PD is a progressive neurodegenerative movement disorder which results due to the loss of dopaminergic (DA) neurons in the basal ganglia [9]. Earlier reports have indicated that microglial activation played a major role in causing damage to DA neurons seen in PD [10]. Therefore, regulation of microglial activation might serve as an important therapeutic strategy in attenuating neuroinflammation.

It has been well reported that triflusal, derived from salicylic acid, was used to treat cardiovascular and inflammatory diseases in Latin America and several European countries for centuries [11]. Earlier reports showed that triflusal and 3-hydroxy-4-trifluoromethylbenzoic acid (HTB) possessed strong antioxidant, anti-inflammatory, and neuroprotective properties based on their cyclooxygenase-2 (COX-2) and nuclear factor kappa beta (NF-κB) inhibitory properties [12]. Metabolic deactylation of triflusal resulted in the generation of HTB, which had the efficacy of antiplatelet drugs, as its parent compound [13]. HTB has also been reported to be effective in inhibiting inflammatory signaling molecules such as cyclic adenosine monophosphate (cAMP), NF-κB, vascular cell adhesion molecule (VCAM)-1, and E-selection [14]. However, both triflusal and HTB bind with the serum proteins of other therapeutic agents such as non-steroidal anti-inflammatory drugs (NSAIDs), warfarin and glisentide, which has limited the use of these agents for combination drug therapy [15,16]. In light of such drawbacks and keeping in mind the activity profile of triflusal and HTB, we synthesized a novel HTB anhydride derivative, namely, 2-hydroxy-4-methylbenzoic anhydride (HMA). In the present study we investigated the inhibitory effects of HMA on neuroinflammation and explored the underlying mechanisms using in vitro lipopolysaccharide (LPS)-stimulated BV-2 microglial cells and an in vivo model of PD based on 1-methyl-4-phenyl-1,2,3,6-tetrahydropyridine (MPTP)-induced neuroinflammation in mice.

## 2. Results

### 2.1. Structure of HMA and Its Effect on Cell Viability and Nitric Oxide (NO) Production in Lipopolysaccharide (LPS)-Induced BV-2 Cells

**Figure 1:**
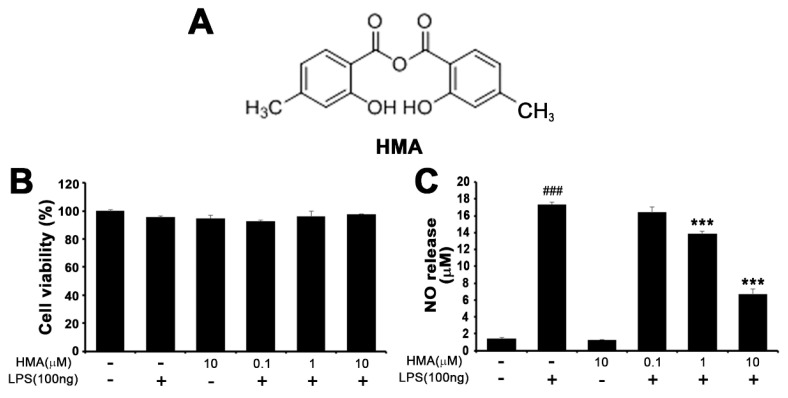
Effect of 2-hydroxy-4-methylbenzoic anhydride (HMA) on the BV-2 microglial cell viability and nitric oxide (NO) release. (**A**) The structural formula of HMA; (**B**) For cell viability, BV-2 cells were pretreated with HMA alone (10 μM) and at various concentrations (0.1, 1.0, and 10 μM) for 1 h, followed by lipopolysaccharide (LPS) treatment (100 ng/mL) for 24 h. Cell viability was measured using the MTT assay; (**C**) NO release was evaluated using culture media in the Greiss assay. Data are mean ± S.E.M. (*n* = 6). ^###^ *p* < 0.001 as compared with control group and *** *p* < 0.001 as compared with LPS-treated group by one-way ANOVA (**C**) F(5,30) = 306.1, *p* < 0.0001)).

The synthesis procedure of HMA is discussed in Methods Section 4.2 and the structure is shown in Figure 1A. To determine whether HMA possessed any effect on overall cell viability, we analyzed the viability in BV-2 microglial cells at various concentrations of HMA with or without LPS (100 ng/mL). Treatment with HMA alone (10 µM) and at various concentrations (0.1, 1, and 10 µM) with LPS did not influence the overall cell viability or show any signs of cytotoxicity as determined by the MTT assay (Figure 1B). For the analysis of nitric oxide (NO) release, accumulated nitrite in the culture media was measured by the Greiss reaction [17]. Production of NO was measured as a standard test to detect inflammatory activation of microglial cells [18,19]. Hence, the suppressive effect of the HMA was evaluated on NO release in LPS-stimulated microglia. In preliminary evaluations, the synthetic HTB derivatives were used to evaluate its suppressive effect on NO release in LPS-stimulated microglia. BV-2 microglial cells were stimulated with LPS for 24 h in the presence of the novel synthetic derivatives (10 µM) or in its absence. In a microglia cell-based assay, a methyl group of R (named HMA, 2-hydroxy-4-methylbenzoic anhydride) was identified as a novel synthetic compound among the synthetic anhydride derivatives of HTB. Among them, three HTB anhydride derivatives with -CH_3,_ -Cl, and -NHBoc of R group showed significant (*p* < 0.001) inhibitory effects of NO release (Supplementary Figure S1B). On the basis of the strong efficacy (more than 70% reduction in NO) exhibited by the -CH_3_ group, we performed further experiments with HMA having -CH_3_ in the R group (Supplementary Figure S1B). Furthermore, HTB strongly attenuated the production of NO without any significant toxicity seen in MTT assay in LPS-stimulated BV-2 microglial cells. In addition, we also compared two HTB derivatives OPTBA (2-((2-oxopropanoyl) oxy)-4-(trifluoromethyl) benzoic acid: HTB-pyruvate ester), and OPMBA (2-((2-oxopropanoyl) oxy)-4-methylbenzoic acid: conversion of HTB-pyruvate ester) with two HTB anhydride derivatives HTBA (2-hydroxy-4-trifluoromethylbenzoic anhydride: HTB anhydride), and HMA (Supplementary Figure S1C). In an earlier report, OPTBA exhibited a neuroprotective effect in the post-ischemic rat brain, which was afforded by anti-excitotoxic and anti-inflammatory effects [20]. Although significant, (*p* < 0.05), the HTB derivatives and HTBA showed lower percentage of inhibition (10–20%) in LPS-induced NO release. However, HMA strongly attenuated NO production (Figure 1C) at the same concentration tested (10 µM). Therefore, further studies were performed on HMA. Stimulation with LPS significantly increased the NO production (*p* < 0.001) as compared with the control group in BV-2 microglial cells. Treatment with HMA alone did not exhibit signs of cytotoxicity in BV-2 microglial cells. However, treatment with HMA at the indicated concentrations (0.1, 1, and 10 µM) in LPS-induced BV-2 cells reduced the NO release to 16.4 ± 0.64 µM, 13.9 ± 0.32 µM, and 6.7 ± 0.63 µM respectively, in a concentration-dependent manner (Figure 1C).

### 2.2. Effect of HMA on the Expression of iNOS and COX-2 on mRNA and Protein Levels in LPS-Induced Microglial BV-2 Cells Formatting of Mathematical Components

**Figure 2:**
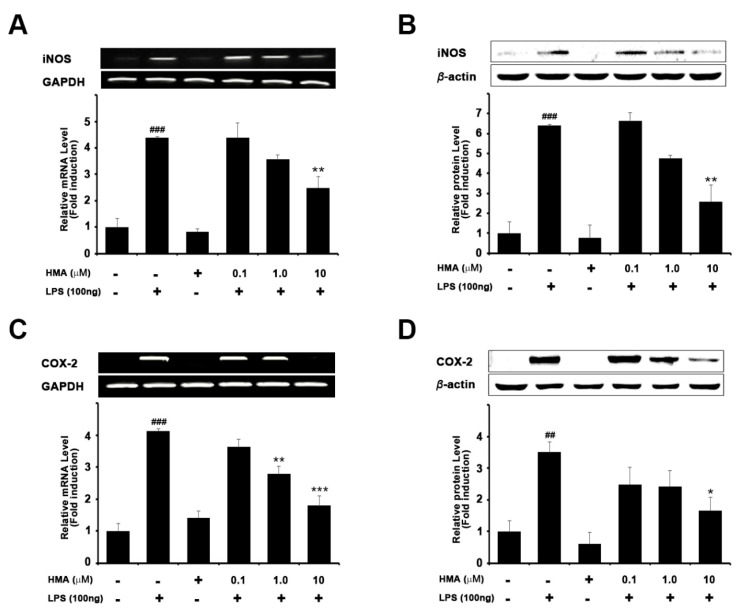
Effect of HMA on iNOS and COX-2 expression in LPS-stimulated BV-2 microglia cells. BV-2 cells were treated with different concentration of HMA (0.1, 1, and 10 µM) for 60 min before incubating with LPS (100 ng/mL) for 6 h (RT-PCR) or 18 h (immunoblot). (**A**,**B**) mRNA and protein expression of iNOS; (**C**,**D**) mRNA and protein expression of COX. Relative expression levels (fold) of iNOS and COX-2 were normalized to that of GAPDH and β-actin for RT-PCR and immunoblotting analysis, respectively. Data are mean ± S.E.M. (n = 3) performed in three independent experiments. ^##^ *p* < 0.01 and ^###^ *p* < 0.001 as compared with the control group; * *p* < 0.05, ** *p* < 0.01, and *** *p* < 0.001 as compared with the LPS-treated group by one-way ANOVA. (**A**) F(5,12) = 23.81, *p* < 0.0001; (**B**) F(5,12) = 24.33, *p* < 0.0001; (**C**) F(5,18) = 31.79, *p* < 0.0001; (**D**) F(5,18) = 6.576, *p* = 0.0012).

The effect of HMA on mRNA and protein levels of iNOS and COX-2 in LPS-stimulated BV-2 microglial cells is shown Figure 2. BV-2 microglia cells were treated with HMA (0.1, 1, and 10 µM) for 1 h, and then stimulated by LPS (100 ng/mL) for another 6 h for polymerase chain reaction (PCR), and 24 h for immunoblotting studies. As shown in Figure 2A,B, LPS-induced BV-2 cells showed a significant (*p* < 0.001) increase in the expression of iNOS. However, HMA treatment, at the indicated concentrations, significantly attenuated increased iNOS mRNA expression levels (4.4- ± 0.56, 3.6- ± 0.18, and 2.5- ± 0.42 fold), and increased iNOS protein levels (6.6- ± 0.42, 4.8- ± 0.14, and 2.6- ± 0.83 fold). A significant reduction was observed with HMA treatment at 10 µM concentration. HMA treatment alone (10 µM) did not influence the iNOS expression. Our data was in agreement with the effect of HMA shown in NO assay.

It is well known that an increase in COX-2 expression plays a significant role in inflammation [3]. Treatment with LPS (100 ng/mL) in BV-2 cells showed a significant increase in COX-2 mRNA (Figure 2C, *p* < 0.001) and protein expression (Figure 2D, *p* < 0.001). However, pretreatment with HMA at various concentrations (0.1, 1, and 10 µM) inhibited LPS-induced COX-2 mRNA expression (3.6- ± 0.22, 2.8- ± 0.24, and 1.8- ± 0.29 fold) and protein levels (2.5- ± 0.53, 2.4- ± 0.49, and 1.7- ± 0.41 fold) in BV-2 microglial cells in a concentration-dependent manner.

### 2.3. Effect of HMA on the Production of the LPS-Stimulated Proinflammatory Cytokines in BV-2 Cells

**Figure 3:**
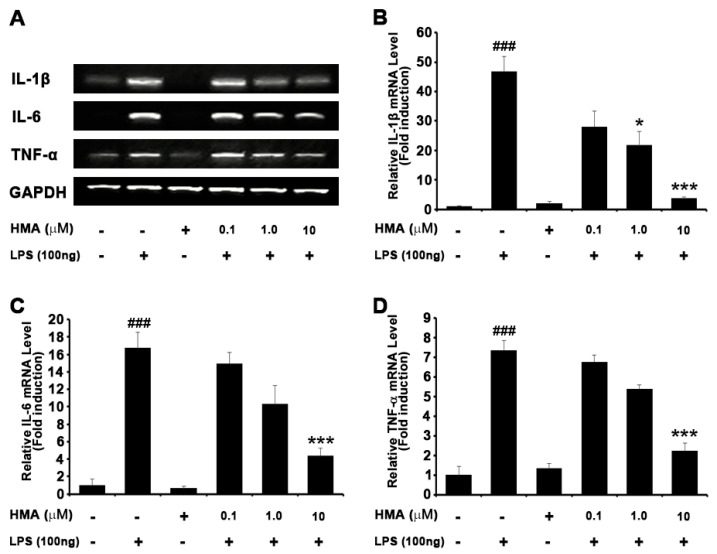
Effect of HMA on proinflammatory cytokines expression in LPS-stimulated BV-2 microglia cells. BV-2 cells were seeded overnight in 6-well plates, and incubated with LPS (100 ng/mL) for 6 h and pretreated with HMA (0.1, 1, and 10 µM) for 1 h. Measurement of the expression level of proinflammatory cytokines (interleukin (IL)-1β, nuclear factor kappa beta (TNF-α), and IL-6) on mRNA level used RT-PCR (**A**) and the corresponding quantification data are shown (**B**–**D**). GAPDH was used as an internal control. Data are presented as the mean ± SEM (*n* = 3) performed in three independent experiments. ^###^ *p* < 0.001 as compared with the control group; * *p* < 0.05 and *** *p* < 0.001 as compared with the LPS-treated group by one-way ANOVA. (**B**) F(5,12) = 10.31, *p* = 0.0005; (**C**) F(5,12) = 18.56, *p* < 0.0001; (**D**) F(5,12) = 25.27, *p* < 0.0001.

To understand the role of HMA on the production of proinflammatory cytokines such as interleukin (IL)-6, IL-1β, and TNF-α, BV-2 microglial cells were stimulated with LPS (100 ng/mL) in the presence of the novel synthetic derivative HMA (0.1, 1, and 10 µM) or in its absence. Following the 6 h of LPS treatment, the RT-PCR analysis showed that the level of IL-6, IL-1β, and TNF-α mRNA expression was increased. Quantification data revealed that pretreatment with HMA (0.1, 1, and 10 µM) for 1 h dose, dependently attenuated the upregulation of IL-1β, IL-6, and TNF-α (Figure 3A). A significant reduction was seen in relative expression of IL-1β (28.1- ± 5.15, 21.7- ± 4.64, and 3.8- ± 0.13 fold), IL-6 (14.9- ± 1.28, 10.3- ± 2.08, and 4.4- ± 0.85 fold), and TNF-α (6.8- ± 0.34, 5.4- ± 0.18, and 2.2- ± 0.36 fold), respectively (Figure 3B–D).

### 2.4. Effect of HMA on NF-κB (p65) Translocation and IκBα Phosphorylation and Degradation

**Figure 4:**
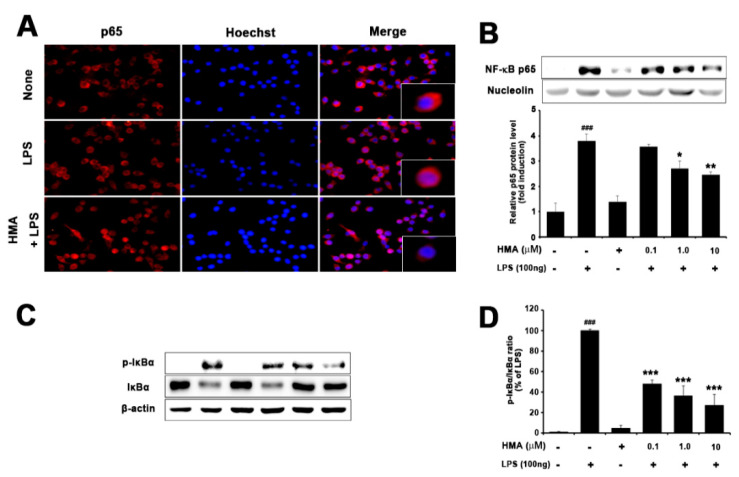
The effect of HMA on inhibition of p65 nuclear factor (NF-κB) translocation, inhibition of IκB-α degradation in LPS- stimulated BV-2 microglial cells. BV-2 cells were seeded overnight in 6-well, 24-well plates, and incubated with LPS (100 ng/mL) for 20 min, and pretreated with HMA (0.1, 1, and 10 µM) for 1 h. (**A**) Translocation of p65 protein was determined using an anti-p65 antibody and Alexa Fluor 568-labeled goat anti-mice antibody on immunofluorescence. The method of Hoechst labelling is described in Section 4.8; (**B**) Isolation of nuclear protein from BV-2 cells, confirmation of translocation quantity of the NF-κB p65 subunit on protein level by Western blot. Nucleolin was used as an internal control; (**C**) Expression of p-IκB-α and IκB-α in LPS-induced BV-2 cells. Total protein from BV-2 cells was extracted and the degradation of p-IκB-α protein level was analyzed by Western blotting, and β-actin was used as an internal control; (**D**) Quantification data of p-IκB-α protein expression (percentage of LPS) normalized by β-actin was shown. Data are presented as the mean ± S.E.M. (*n* = 3) performed in three independent experiments. ^###^ *p* < 0.001 as compared with the control group; * *p* < 0.05, ** *p* < 0.01, and *** *p* < 0.001 as compared with the LPS-treated group by one-way ANOVA. (**B**) F(5,12) = 21.80, *p* < 0.0001 and (**D**) F(5,12) = 22.50, *p* < 0.0001.

According to previous studies, degradation of IκB-α through phosphorylation and the subsequent nuclear translocation of p65 subunit into nucleus is necessary for activation of NF-κB [21,22]. The NF-κB signaling pathway is activated by LPS-induced proinflammatory cytokines at an early state in BV-2 microglial cells. Moreover, NF-κB plays a significant role in LPS-induced expression of iNOS, COX-2, and proinflammatory cytokines. Therefore, to understand the mechanism of HMA, we examined the translocation of p65 and degradation of IκB-α in LPS-stimulated BV-2 cells. As shown in Figure 4A, the immunofluorescence assay revealed that HMA regulated the translocation of the NF-κB subunit p65 into the nucleus. For further characterization of the mechanism by which HMA inhibited the proinflammatory cytokines expression, Western blotting was conducted to see the effect of HMA on blocking of NF-κB/p65 translocation and IκB-α phosphorylation in BV-2 cells (Figure 4B,C). Quantification data revealed that HMA treatment significantly attenuated the IκB-α degradation in LPS-stimulated BV-2 microglial cells (Figure 4D).

### 2.5. HMA Shows the Protective Effect of the Behavioral Deficit against MPTP Toxicity in a Mouse Model of Parkinson’s Disease (PD): The Pole Test

**Figure 5:**
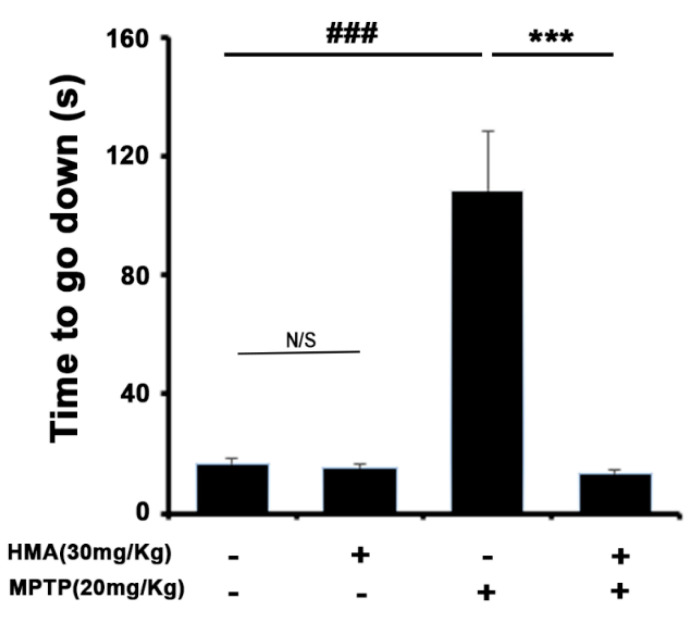
Effect of HMA on the behavioral deficit in 1-methyl-4-phenyl-1,2,3,6-tetrahydropyridine (MPTP)-intoxicated mouse. Thirty mg/kg, p.o. (HMA) was dosed for 15 days before MPTP administration (20 mg/kg, 4 injections/day, 2 h interval, i.p.). Values are mean ± S.E.M, *n* = 12. N/S (not significant) and ^###^ *p* < 0.001 as compared with control group; *** *p* < 0.001 as compared with MPTP-intoxicated group by one-way ANOVA. (F(3,44) = 21.23, *p* < 0.0001).

A pole test was performed to evaluate the bradykinesia in MPTP-intoxicated mice. The pole test results indicated a significantly prolonged total locomotor activity (TLA) after MPTP treatment. The time to reach the platform was significantly shortened after the prophylactic treatment with HMA at 30 mg/kg (Figure 5). The group treated with HMA alone did not show any effect on the TLA and was normal.

### 2.6. Effect of HMA on Microglial and Glial Activation in MPTP-Intoxicated Mouse Model of PD

**Figure 6:**
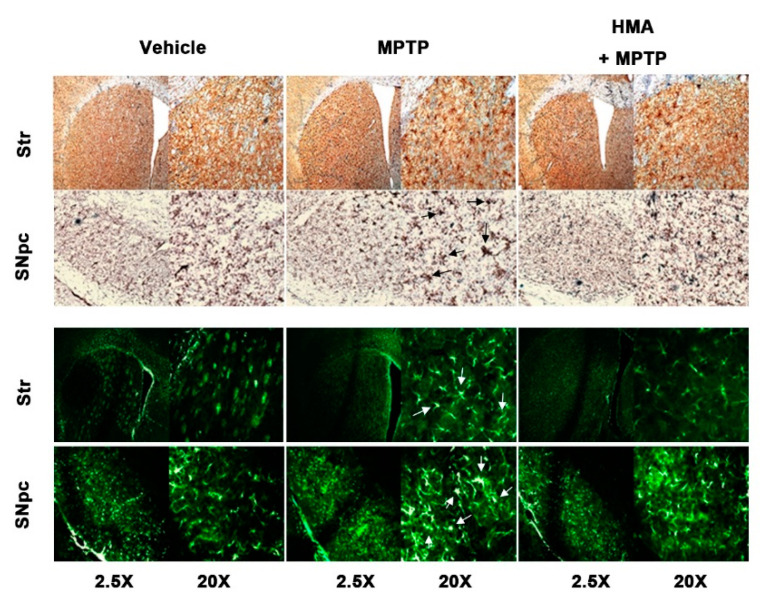
Inhibitory effect of HMA on MPTP-induced activated microglial and glial markers expression in the striatum (STR) and substantia nigra pars compacta (SNpc). Representative microphotographs of macrophage Ag complex-1 (MAC-1, upper panel) and glial fibrillary acidic protein (GFAP, lower panel) by immunohistochemistry-immunofluorescence assay (IHC-IF). HMA (30 mg/kg) was administered for 14 days and, MPTP was administered 20 mg/kg (four injections per day at 2 h intervals) on the last day of HMA treatment. Mice were anesthetized for the IHC-IF study 1 day after the last MPTP treatment. The activation sites in MPTP-induced mouse brain tissues are indicated with arrows.

**Figure 7:**
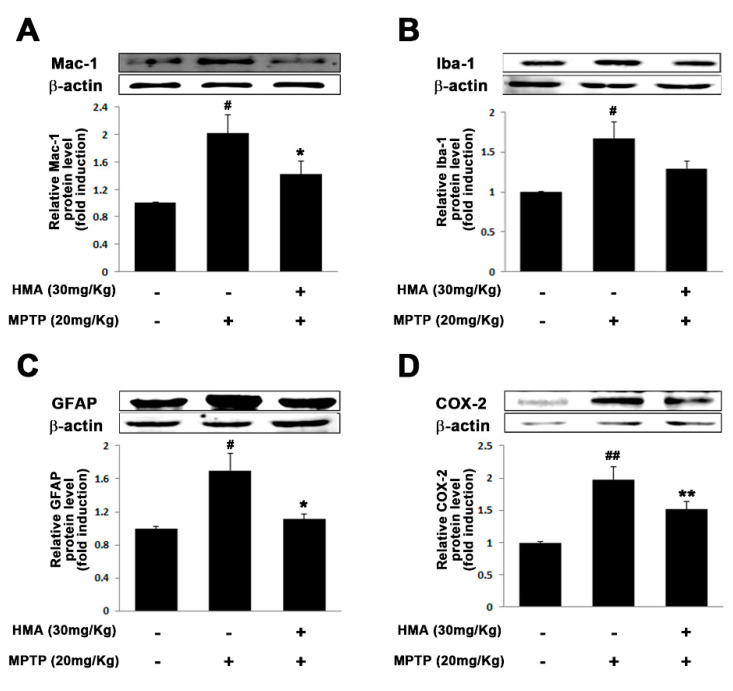
Effect of HMA on MAC-1, Iba-1, GFAP, and COX-2 expressions in MPTP-intoxicated mice. MAC-1 (**A**), Iba-1 (**B**), GFAP (**C**) and COX-2 (**D**) protein levels in the SNpc were assessed by Western blot analysis. Corresponding relative expression (fold change) levels are shown, respectively. β-Actin was used as an internal control. Data are mean ± S.E.M. (*n* = 3) performed in three independent experiments. ^#^ *p* < 0.05 and ^##^ *p* < 0.01 as compared with the control group; * *p* < 0.05 and ** *p* < 0.01 as compared with the MPTP-intoxicated group by one-way ANOVA. (**A**) F(2,6) = 6.609, *p* = 0.304; (**B**) F(2,6) = 5.835, *p* = 0.391; (**C**) F(2,6) = 6.788, *p* = 0.0288; (**D**) F(2,6) = 24.33, *p* = 0.0013.

Activation of microglia followed by MPTP intoxication is a well-known pathological feature in rodents [22]. To assess the preventive effect of HMA on both microglial and glial activation followed by MPTP-intoxicated mouse model of PD, we detected macrophage Ag complex (MAC)-1 and glial fibrillary acidic protein (GFAP) as microglial and glial markers, respectively, with anti-MAC and anti-GFAP antibodies. The IHC-IF assay revealed a significant increase in the number of activated microglia in the substantia nigra pars compacta (SNpc) and striatum of MPTP-intoxicated mice as compared with vehicle and HMA-treated groups (Figure 6). In addition, an increase in microglial activation-related markers such as MAC-1 and Iba-1 protein was also observed in the SNpc as a result of MPTP intoxication (Figure 7A,B). MPTP also increased the GFAP protein expression (Figure 7C) in the SNpc. After HMA treatment, this microglia activation was prevented, which was visible in MAC-1 and Iba-1 expression in the SNpc (Figure 7A,B). The increased GFAP expression was also suppressed when treated with HMA (Figure 7C). Furthermore, to evaluate whether or not HMA had an effect on the COX-2 protein expression in MPTP-intoxicated mice, Western blot analysis was performed. Data showed that the COX-2 protein expression level was increased after one day of MPTP treatment. However, HMA treatment significantly attenuated the increased expression of COX-2 (Figure 7D). These results indicated that HMA exerted its action by preventing the COX expressions. A significant reduction was seen in the relative signal intensity in the densitometry analysis of the bands (lower panels).

## 3. Discussion

In this study, we used in vitro and in vivo experimental models to evaluate a novel HTB anhydride derivative, HMA, and investigated its inhibitory effects on neuroinflammation at significantly lower concentrations as compared with its parent compounds. To date, BV-2 microglial cells stimulated by LPS remain to be a classical in vitro model to study the inflammatory mediated mechanisms and to screen several new anti-inflammatory agents [23]. Reports have also shown that, after treatment with LPS, many neurotoxic factors were produced in brains through microglial activation. Similarly, after the administration of MPTP, it produced irreversible and severe Parkinsonian syndrome-like behavioral impairments in both humans and experimental animals [24]. Furthermore, an increase in immune molecules and early microglial activation after MPTP intoxication has also been reported [25]. Therefore, stimulation of microglial cells by LPS in vitro and MPTP-intoxicated mouse model of PD in vivo are considered to be standard valid models for investigating agents inhibiting neuroinflammation [26,27]. In the current study, we used these two models to evaluate the effect of HMA and the underlying mechanisms.

Microglial cells are one of the first lines of defense used by the brain against a chemical or mechanical injury [28]. In pathological conditions, microglial cells can produce free radicals, chemokines, and proinflammatory cytokines, which accelerate neuroinflammation, causing neuronal toxicity and neurodegeneration [3]. In the present investigation, HMA strongly inhibited the microglia-mediated inflammation. HMA inhibited NO release by suppressing both mRNA and protein expression of iNOS. It has been reported that activated microglia could increase the expression of COX-2 and its expression was pathogenic in the progression of PD [29,30]. In this study, HMA significantly inhibited the increased mRNA and protein expression of COX-2 in LPS-induced BV-2 cells. Furthermore, increased levels of TNF-α, IL-6, and IL-1β proinflammatory cytokines have been reported to play a central role in neuronal damage mediated by inflammation. Their production was elevated during the activation of microglia. The transcription factor NF-κB signaling played a pivotal role in innate and adaptive immunity [31,32]. In addition, NF-κB has been reported to control inflammatory responses through regulating the expression of proinflammatory cytokines such as TNF-α, IL-6, IL-1β, COX-2, and iNOS in activated microglia cells [3,10]. The phosphorylation and subsequent degradation of IκB has been found to trigger the activation of NF-κB. Thus, the inhibition of NF-κB and resultant release of proinflammatory elements were advantageous to suppress microglia-mediated neuroinflammation [33]. Here, HMA suppressed the production of LPS-induced proinflammatory mediators significantly and inhibited the activation of NF-κB by blocking the IκB-α degradation, which indicated that HMA could be beneficial to suppress the progression of neuroinflammation in many neurodegenerative disorders including PD.

MPTP intoxication has been reported to create behavioral deficits in mice [34,35,36]. In addition, in PD, DA neuronal cell has been reported to be a result of inflammation-associated factors released in response to microglial activation [10,37,38,39]. Moreover, activated microglia potentiate the upregulation of the expression of cell surface markers such as MAC-1 and GFAP [40,41,42]. In view of the published reports, in our present study, MPTP intoxication in mice produced behavioral deficits and prolonged TLA in the pole test, and decreased locomotor activities. The protective effect of HMA to mitigate bradykinesia, as compared with the only MPTP treated group, was revealed by the pole test. Groups treated with HMA alone did not show an increase or decrease in locomotor activity at the indicated dose. However, prophylactic treatment with HMA (30 mg/kg, p.o.) significantly reversed these events in the MPTP-intoxicated groups. Furthermore, the protein expression of cell surface markers, MAC-1, Iba-1, GFAP, and COX-2 in the SNpc was upregulated, following the intoxication of MPTP in mice which were significantly inhibited by HMA treatment. On the basis of the overall results, our present investigation clearly indicated that HMA attenuated the in vitro microglia-mediated neuroinflammatory processes and in vivo MPTP-intoxicated behavioral deficits in mouse PD model.

## 4. Materials and Methods

### 4.1. Materials

LPS (Escherichia coli 0111: B4, Sigma, St. Louis, MO, USA), MPTP, N-(1-naphthyl) ethylenediamine dihydrochloride, and 3-(4,5-dimethylthiazol-2-yl)-2,5-diphenyltetrazolium bromide (MTT), bovine serum albumin (BSA), Tween-20, dimethyl sulfoxide (DMSO) (compound diluent/(1%) vehicle control), sulfanilamide, and sodium nitrite were purchased from Sigma (St. Louis, MO, USA). Dulbecco’s modified Eagle’s medium (DMEM), phosphate buffered saline (PBS) and other culture reagents were purchased from Gibco/Invitrogen (Carlsbad, CA, USA). Fetal bovine serum (FBS) was purchased from PAA Laboratories Inc. (Etobicoke, Ontario, Canada). The cocktail tablets of the protease and phosphatase inhibitor were supplied by Roche (Indianapolis, IN, USA). Antibodies to iNOS, phospho-p38, p38, phospho-IκB-α, IκB-α, and β-actin were supplied by Cell Signaling Technology (Danvers, MA, USA) and antibodies to COX-2, p65 NF-κB, and nucleolin were supplied from Santa Cruz Biotechnology (Santa Cruz, CA, USA).

### 4.2. Synthesis of 2-Hydroxy-4-Methylbenzoic Anhydride (HMA)

HMA was synthesized by dissolving 2-hydroxy-4-methylbenzoic acid (2.00 g, 9.70 mmol) in tetrahydrofuran (THF) (150 mL), and dicyclocarbodiimide (DCC) (2.71 g, 13.1 mmol) was added to this solution, followed by stirring at room temperature for 24 h. The formed solid was filtered off and the residue was evaporated in vacuo. The title compound was produced as a white solid (560 mg, 31%) after the crude product was purified by silica gel column chromatography. Mp 171 °C; 1H NMR (CDCl3) δ 2.44 (s, 6H), 7.18–7.22 (d, 1H, J = 8.0 Hz), 7.31 (s, 2H), 7.82–7.86 (d, 2H, J = 8.0 Hz); 13C NMR (CDCl3) δ 21.81, 120.86, 124.17, 126.77, 131.50, 144.31, 148.40, and 164.52. The detailed synthesis procedure of HMA is shown in Supplementary Figure S1A.

### 4.3. Cell Culture and Treatment

The BV-2 microglial cells were obtained as described previously [34]. Briefly, cells were cultured and maintained in DMEM supplemented 5% FBS and 50 μg/mL penicillin-streptomycin in a humidified incubator at 37 °C with 5% CO_2_. The seeded in density of BV-2 cells was 1 × 10^6^ cells/mL. Cells were treated, for 1 h, with synthetic HTB derivatives and different concentrations of HMA (0.1, 1, and 10 µM), prior to be incubated with LPS (100 ng/mL) for 30 min, and 6, 18, and 24 h.

### 4.4. Animals and Treatment

Male C57BL/6 mice aged 6 weeks, and weight 25–28 g, were supplied from the Samtako Bio Korea (Gyeonggi-do, Korea). Before using them in the study, all mice were acclimatized to laboratory conditions for 2 weeks. All experiments were performed in accordance with the Principles of Laboratory Animal Care (NIH publication no. 85-23, revised 1985) and Guidelines for the Institutional Animal Care and Use Committee of Konkuk University (no. KU17024, 15/02/2017). The animals were housed in a controlled environment, maintained at 23 ± 1 °C temperature and 50% ± 5% humidity, and food and water were supplied ad libitum. The room lights were turned on 12 h between 8:00 and 20:00. All mice were divided into three different groups, each containing 12 mice (36 in total). The groups included a vehicle group, MPTP 20 mg/kg-induced group (four times, 1 day at 2 h intervals, i.p.) and HMA 30 mg/kg (14 days, p.o.) + MPTP 20 mg/kg (four times, 1 day at 2 h intervals, i.p.) group. HMA was administered orally for 14 days, whereas MPTP was administered i.p. on the 14th day of HMA treatment. The HMA and MPTP solutions were both prepared in saline just prior to administration. The safety measures of MPTP is based on ”Protocol for the MPTP mouse model of Parkinson’s disease” [43].

### 4.5. Cell Viability and NO Assay

The 2.5 × 10^4^ cells/mL BV-2 cells were seeded in 96-well plate. Cells were pretreated with indicated synthetic HTB derivatives for 1 h, with or without LPS (100 ng/mL) for 24 h. The effect of HMA on BV-2 cell viability was determined, as described previously [34]. After treatment for the desired time, supernatants were suctioned or removed by flipping the plate. The formazan crystals formed in viable cells were dissolved in DMSO. Optical density was measured at 550 nm using a microplate reader (Tecan Trading AG, Basel, Switzerland) and the values were compared with untreated cells.

The inhibitory effect of synthetic HTB derivatives and HMA (0.1, 1, and 10 µM) on nitrite concentration was determined, as described previously [44]. Briefly, a standard curve was generated using a range of dilutions of known concentration of sodium nitrite. Approximately 540 nm wavelength was used to measure the absorbance in a microplate reader (Tecan Trading AG).

### 4.6. Total RNA Extraction and RT-PCR

In 6-well plates, BV-2 cells at a density of 5 × 10^5^ cells/mL were seeded, and total RNA was isolated by using TRIzol reagent (Invitrogen). Total 2.5 µg of RNA was reverse transcribed for RT-PCR using a First-Strand cDNA Synthesis kit (Invitrogen). Then, using specific primers, cDNA was amplified by PCR following the method described previously in [45]. The synthesized cDNA was further used as a template to perform PCR for respective targets; 1% agarose gels were used to analyze PCR products.

### 4.7. Western Blot Analysis

Cells were washed twice with ice cold PBS, and total cell lysates were obtained by adding 50 or 100 mL of RIPA buffer (PBS, 1% NP-40, 0.5% sodium deoxycholate, and 0.1% SDS, containing fresh protease inhibitor cocktail) to the BV-2 cells (5 × 10^5^ cells/mL) cultured in 6-well plates. The Bio-Rad DC Protein Assay was performed to determine the protein concentration (Hercules, CA, USA). Then, 10% sodium dodecyl sulfate-polyacrylamide electrophoresis (SDS-PAGE) was used to separate 40 µg of whole cell lysates (in vitro experiments) electrophoretically. The resolved proteins were transferred to polyvinylidene difluoride (PVDF) membranes (Millipore, Bedford, MA, USA). To block nonspecific binding, the PVDF membranes were incubated for 1 h, with 5% skim milk in PBS buffer. PVDF membranes were, then, incubated overnight with anti-iNOS (1:1000), anti-COX-2 (1:1000), anti-IκB-a (1:1000), anti-p-IκB-a (1:1000), anti-p38 (1:1000), anti-p-p38 (1:1000), anti-p-p65 NF-κB (1:500), anti-β-actin (1:2000), and anti-nucleolin (1:500) antibodies, followed by a 1 h incubation with horseradish peroxidase-conjugated secondary antibody (1:1000–2000). A LAS-3000 Luminescent Image Analyzer (Fuji, Tokyo, Japan) was used to analyze the optical densities of the antibody specific bands. In a parallel experiment, nuclear proteins were extracted using the method provided by the nuclear and cytoplasmic extraction kit from Thermo Scientific (Rockford, IL, USA).

### 4.8. Immunocytochemistry

To detect the intracellular location of the NF-κB p65 subunit, BV-2 cells were cultured at a density of 5 × 10^5^ cells/well on sterile 12 mm cover slips in a 24-well plate and treated with HMA (10 μM) and LPS (100 ng/mL). Twenty min after the LPS treatment, the cells were fixed with methanol for 20 min at −20 °C, and washed with PBS for 5 min. After fixing the cells, they were permeabilized for 10 min in room temperature with 1% Triton X-100 in PBS, and then washed with PBS for 5 min, followed by incubation with 10% goat serum in PBS for 60 min. The permeabilized cells were, then, treated with monoclonal mice anti-human NF-κB (p65) (1:200) (Santa Cruz Biotechnology) for 60 min at room temperature and washed with PBS for 5 min. The cells were, then, incubated in a 1:200 dilution of Alexa Fluor 568-labeled goat anti-mice antibody (Invitrogen) for 60 min at room temperature and washed in PBS for 5 min. Cells were stained with 1 μM Hoechst staining solution for 7 min at 37 °C, and then washed. Finally, the cover slips with cells were dried at 55 °C in an oven, for 15 min, and mounted in a 1:1 mixture of xylene and malinol. More than 50 cells per field were counted under a fluorescence microscope (Carl Zeiss Inc., Oberkochen, Germany).

### 4.9. Immunohistochemistry

Following the behavioral experiments, sodium pentobarbital (50 mg/kg, i.p.) was used to anesthetize experimental animals treated with MPTP or vehicle/HMA for immunohistochemical-immunofluorescence (IHC-IF) evaluations. The brain of each mouse was collected, perfusion-fixed with 4% paraformaldehyde in 0.1 M phosphate buffer (pH 7.4) following a saline flush, as described previously [36,45]. Stained cells were viewed using a bright filed microscope (Carl Zeiss).

### 4.10. Pole Test

To measure bradykinesia, a pole test was conducted, using a slight modification of a previously described method [36,45]. Total locomotor activity (TLA) was measured by positioning a mouse upward at the top of a rough-surfaced pole which was 8 mm in diameter and 55 cm in height. TLA was the time until the mouse arrived at the floor. The delay in the time required to complete the test was considered to reflect bradykinesia. The pole test was performed five times successively for each mouse.

### 4.11. Statistical Analyses

Data are expressed as mean ± standard error of mean (S.E.M). Graph Pad Prism version 5.01 (Graph Pad, Inc., La Jolla, CA, USA) was used to analyze the data. Comparisons between the control and experimental groups were made using a one-way analysis of variance (ANOVA) followed by Dunnett’s multiple comparison tests. *p*-values < 0.05 were considered statistically significant.

## 5. Conclusions

HMA exhibited strong inhibitory effects in regulating neuroinflammation in cellular and experimental animal models of PD in significantly lower concentrations (up to 10 μM in vitro) than its parent compounds. The significant beneficial effects showed by HMA could partly be mediated via regulation of the NF-κB signaling pathways. Supporting the in vitro data, HMA also attenuated the behavioral deficits and suppressed the microglial activation in an in vivo mouse model of PD. However, further work is warranted in other animal and genetic models of PD to understand the detailed mechanism involved for evaluating the efficacy of HMA, to develop a potential therapeutic agent in the treatment of microglia-mediated neuroinflammatory disorders, including PD.

## Supplementary Materials

Supplementary Materials can be found at https://www.mdpi.com/1422-0067/21/21/8195/s1. Figure S1. Synthesis of HTB and Evaluation of the reduction of NO secretion of synthetic HTB anhydride derivatives in LPS-treated BV-2 microglial cells.
