# Supplementary material for: 2-Hydroxy-4-Methylbenzoic Anhydride Inhibits Neuroinflammation in Cellular and Experimental Animal Models of Parkinson’s Disease
Source: Int J Mol Sci. 2020 Nov 2;21(21):8195. doi: 10.3390/ijms21218195 (PMC7662568; doi:10.3390/ijms21218195)
Supplement: Supplementary file 1 [file ijms-21-08195-s001.pdf]

2-hydroxy-4-methylbenzoic anhydride (HMA) attenuates microglia-mediated neuroinflammatory responses in in vitro activated microglia and in vivo experimental models of Parkinson's disease

Soo-Yeol Song[1#], In- Su Kim[1#], Sushruta Koppula[1], Ju-Young Park[2], Byung-Wook Kim[1], Sung-Hwa Yoon[2], Dong- Kug Choi[1*]

[1] Department of Biotechnology, Konkuk University, Chungju 380-701, Korea ; go_world87@nate.com, kis5497@kku.ac.kr, koppula@kku.ac.kr, kbwxfile@nate.com

[2] Department of Molecular Science and Technology, Ajou University, Suwon 443–749, Korea ; pink1209juyoung@empas.com, shyoon@ajou.ac.kr

* Correspondence: choidk@kku.ac.kr; Tel.: +82-43-840-3616

[#] Both authors contributed equally to this work.

Materials and Methods

Cell culture

The BV-2 microglial cells were obtained as described previously [34]. Briefly, cells were cultured and maintained in DMEM supplemented 5% FBS and 50 μg/mL penicillin–streptomycin in a humidified incubator at 37 °C with 5% CO2.

NO assay

2.5 × 10^4 cells/mL BV-2 cells were seeded in 96-well plate. Cells were pre-treated with indicated synthetic HTB derivatives for 1h with or without LPS (100 ng/mL) for 24 h.The inhibitory effect of synthetic HTB derivatives on nitrite concentration was determined as described previously [1]. Briefly, a standard curve was generated using range of dilutions of known concentration of sodium nitrite. Approximately 540 nm wavelength was used to measure the absorbance in a microplate reader (Tecan Trading AG).

Results

In a microglia cell-based assay, methyl group of R (named HMA; 2-hydroxy-4-methylbenzoic an hydride) was identified as a novel synthetic compound among the synthetic anhydride derivativ es of HTB. Among them, three HTB anhydride derivatives with -CH_3, -Cl and -NHBoc of R gro up showed significant ($p < 0.001$) inhibitory effects of NO release (sup. Fig. 1B). Based on the st rong efficacy (more than 70 % in reduction of NO) exhibited by -CH_3 group, we performed furt her experiments with HMA having -CH_3 in the R group (sup. Fig. 1B). Further, HTB strongly at tenuated the production of NO without any significant toxicity seen in MTT assay in LPS-stimu lated BV-2 microglial cells. Besides, we also compared two HTB derivatives OPTBA (2-((2-oxopr opanoyl) oxy)-4-(trifluoromethyl) benzoic acid: HTB-pyruvate ester), and OPMBA (2-((2-oxopropa noyl) oxy)-4-methylbenzoic acid: conversion of HTB-pyruvate ester) with two HTB anhydride d erivatives HTBA (2-hydroxy-4-trifluoromethylbenzoic anhydride: HTB anhydride), and HMA (su p. Fig. 1C). Although significant, ($p<0.05$), the HTB derivatives and HTBA showed lower percen tage of inhibition (10-20 %) in LPS-induced NO release. However, HMA strongly attenuated N O production at the same concentration tested (10 μM) (sup. Fig. 1C).

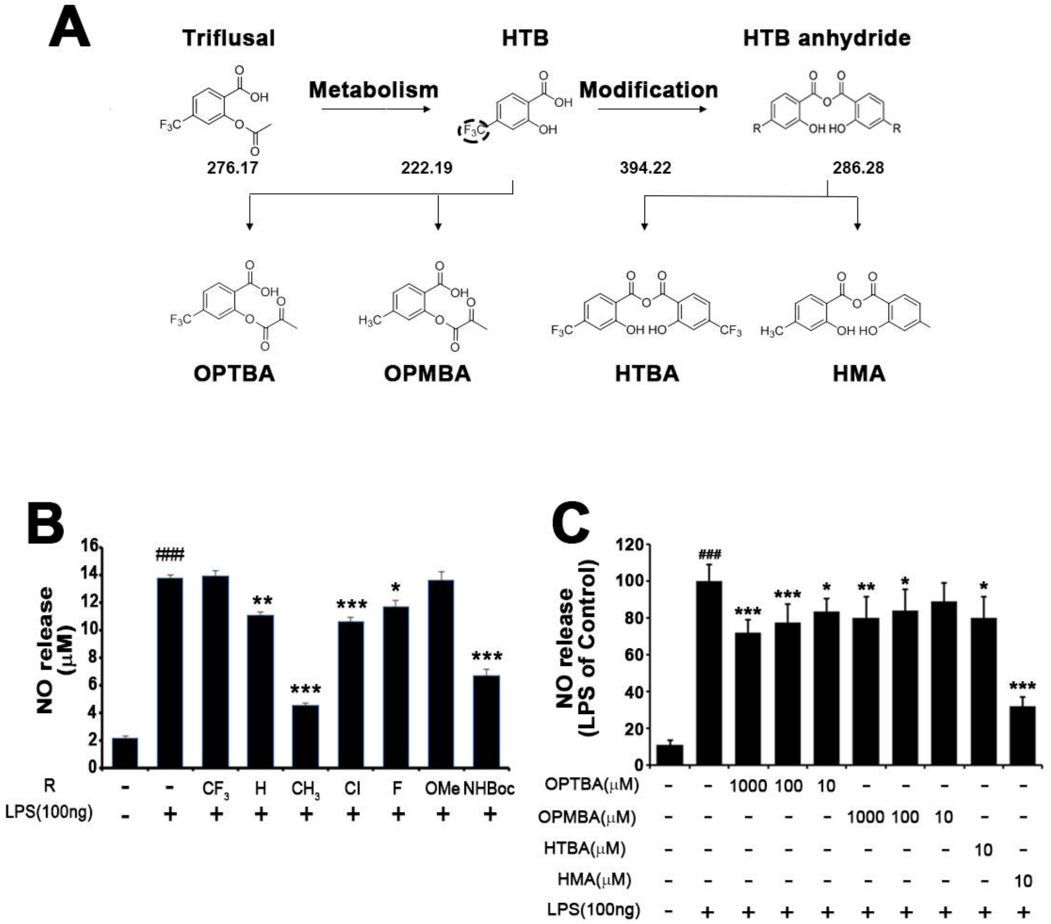

Figure S1. Synthesis of HTB and Evaluation of the reduction of NO secretion of synthetic HTB anhydride derivatives in LPS-treated BV-2 microglial cells. The detailed synthetic procedure is shown (A). (B) The effect of HTB anhydride derivatives on nitric oxide (NO) production and cytotoxicity in lipopolysaccharide (LPS)-stimulated BV-2 microglial cells. BV-2 microglial cells were treated with LPS (100 ng/ml) in the absence or presence of the novel synthetic HTB anhydride derivatives (10 μM) for 24 h. (C) BV-2 cells were pretreated with two HTB derivatives (OPTBA, OPMBA) (1000, 100, 10 μM) and two HTB anhydride derivatives (HTBA, HMA)(10 μM) for 1 h, followed by LPS treatment (100 ng/mL) for 24 h. NO release was evaluated using culture media in the Griess assay (B,C) Data are mean ± S.E.M. (n=8). ###P < 0.001, compared with control group; *P<0.05, **P < 0.01 and ***P < 0.001 compared with LPS alone group by One-way ANOVA.

References

1. Kim, B. W.; Koppula, S.; Kim, J. W.; Lim, H. W.; Hwang, J. W.; Kim, I. S.; Park, P. J.; Choi, D. K., Modulation of LPS-stimulated neuroinflammation in BV-2 microglia by Gastrodia elata: 4-hydroxybenzyl alcohol is the bioactive candidate. *J Ethnopharmacol* **2012,** 139, (2), 549-57.
